# THQ–Xanthene: An Emerging Strategy to Create Next‐Generation NIR‐I/II Fluorophores

**DOI:** 10.1002/advs.202301177

**Published:** 2023-04-28

**Authors:** Zhiqiang Mao, Hyeonji Rha, Jungryun Kim, Xinru You, Fan Zhang, Wei Tao, Jong Seung Kim

**Affiliations:** ^1^ College of Health Science and Engineering College of Chemistry and Chemical Engineering Hubei University Wuhan 430062 China; ^2^ Department of Chemistry Korea University Seoul 02841 South Korea; ^3^ Center for Nanomedicine and Department of Anesthesiology Brigham and Women's Hospital Harvard Medical School Boston MA 02115 USA

**Keywords:** fluorescent imaging, near‐infrared I/II, rhodamine, theranostic, xanthene

## Abstract

Near‐infrared fluorescence imaging is vital for exploring the biological world. The short emissions (<650 nm) and small Stokes shifts (<30 nm) of current xanthene dyes obstruct their biological applications since a long time. Recently, a potent and universal THQ structural modification technique that shifts emission to the NIR‐I/II range and enables a substantial Stokes shift (>100 nm) for THQ‐modified xanthene dyes is established. Thus, a timely discussion of THQ–xanthene and its applications is extensive. Hence, the advent, working principles, development trajectory, and biological applications of THQ–xanthene dyes, especially in the fields of fluorescence probe‐based sensing and imaging, cancer theranostics, and super‐resolution imaging, are introduced. It is envisioned that the THQ modification tactic is a simple yet exceptional approach to upgrade the performance of conventional xanthene dyes. THQ–xanthene will advance the strides of xanthene‐based potentials in early fluorescent diagnosis of diseases, cancer theranostics, and imaging‐guided surgery.

## Graphical abstract

THQ modification strategy emerges as an efficient approach to endow xanthene dyes with NIR‐I/II emissions (650–1700 nm) and remarkable Stoke shifts (100 nm). Herein, the advent, working principles, chemical structural modifications, and evolutional trajectory of THQ–xanthene dye‐based imaging and sensing, theranostics, and super‐resolution imaging applications, are systematically introduced.

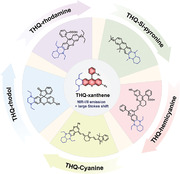

## 1 Introduction

Near‐infrared (NIR) fluorescence imaging has been an indispensable approach to exploring biological and life processes.^[^ ^1^ ^]^ Based on the excitation and emission wavelengths, the NIR imaging territories can be roughly sorted into two categories: NIR‐I (650–900 nm) imaging and NIR‐II (900–1700 nm) imaging.^[^ ^2^ ^]^ It is well‐documented that fluorescence signals at the NIR‐I or NIR‐II windows have better imaging penetration depths in tissues and suffer from less scattering and background fluorescence to get high‐fidelity visualization in comparison with ultraviolet to visible‐light (<650 nm) imaging. NIR‐I/II imaging is much more suitable for living body imaging, such as drosophila, zebrafish, mice, rabbits, and monkeys.^[^ ^3^ ^]^ Nevertheless, the footing stone for NIR‐I/II imaging is NIR‐I/II emissive fluorophores/dyes with fabulous optical performances. To date, NIR‐I/II fluorophores are constructed from molecular fluorophores, fluorescent proteins (FPs), luminescent nanomaterials, and so on.^[^ ^4^ ^]^ In contrast to FPs and luminescent nanomaterials, molecular dyes are superior and triumphant in favorable biocompatibility, easy operation, and structure‐tunable NIR emission properties. For these reasons, NIR molecular dyes have been broadly utilized in fluorescent sensing and imaging in vivo, bacteria‐killing, cancer therapy, and dye‐sensitized solar cells.^[^ ^5^ ^]^

Xanthene dyes embrace rhodamine, fluorescein, rhodol, and other xanthene‐like dyes, and they share large absorption coefficients, good water‐solubility, low cytotoxicity, and high fluorescence quantum yields.^[^ ^6^ ^]^ In 1871, von Bayer originally synthesized fluorescein, and 18 years later, in 1889, Frieds first synthesized the rhodol. In 1905, Noelting and Dziewonsky reported Rhodamine for the first time.^[^ ^7^ ^]^ With more than one century's development, xanthene dyes have been widely harnessed in fluorescence assay, fluorescence/photoacoustic bioimaging, industrial dyes, and photodynamical and photothermal therapy (PDT/PTT).^[^ ^8^ ^]^ Compared with conventional NIR dyes, such as cyanine, semi‐cyanine, BODIPY, and squaraine, in vivo imaging and phototherapeutic applications of xanthene dyes were far from satisfactory for a long time in terms of the short excitation/emission of these dyes.

**Scheme 1:**
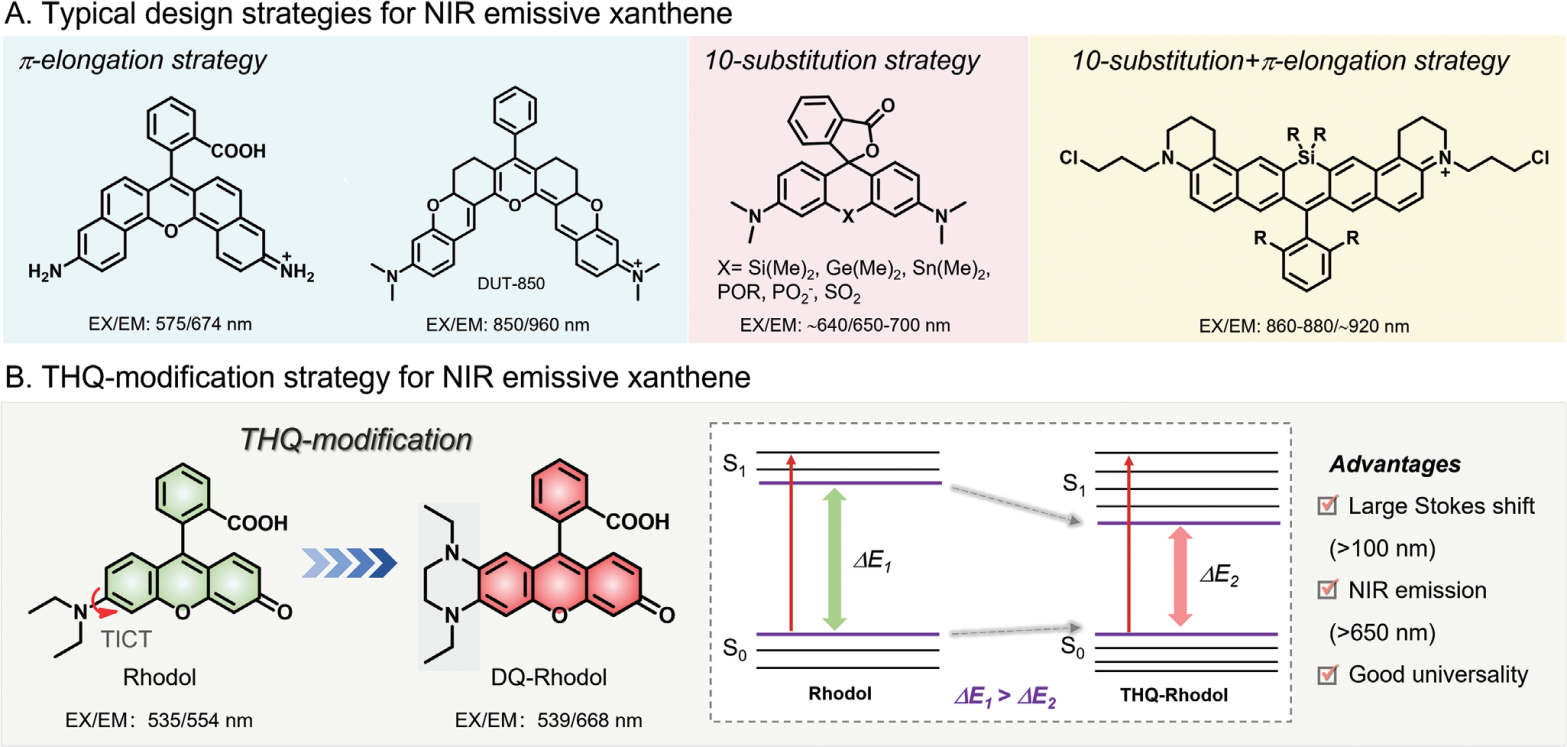
Chemical design of THQ–xanthene. A) Conventional design strategies for NIR emissive xanthene. B) The THQ‐modification strategy to design NIR xanthene dyes (Take THQ–rhodol as an example).

To cope with the awkward situation of xanthene dyes, a bolus of elegant research has been reported, proposing to upgrade the excited/emissive wavelengths and brightness in the NIR‐I/II range for the past decades. The classic and robust method to extend the emission wavelengths is by increasing the *π*‐conjugation of original xanthene dyes, named the *π*‐elongation strategy (**Scheme** **1**A).^[^ ^9^ ^]^ For example, naphthalene rings were incorporated with xanthene scaffolds (rhodamine, fluorescein, and rhodol) and their *π*‐conjugation was expanded to get NIR emissive xanthene dyes, such as naphthorhodamine, naphthofluorescein, and naphthorhodol.^[^ ^10^ ^]^ Further, in 2017, Yang's group developed bisbenzo‐C‐rhodamine derived ECX dyes that featured absorption and emission centered at some 880/915 nm, high fluorescence quantum yields (13.3%), and high photo/chemo‐stability.^[^ ^11^ ^]^ By taking a similar tactic, in 2022, Ma et al. reported novel xanthene‐based NIR‐II dyes by simply coupling two styryls (hydroxystyry, methoxystyryl, *N,N*‐dimethylstyryl, and julolidinestyryl) to the 3,6‐positions of fluorescein core, with an unprecedentedly maximal emission at 1210 nm, enabling the measurement of in vivo blood flow in the artery and vein at NIR‐II window.^[^ ^12^ ^]^ To tackle the strong hydrophobicity and *π*–*π* stacking‐induced quenching of *π*‐elongated xanthene dyes, in 2022, Xiao et al. created a naphthorhodamine‐evolved V‐shaped NIR‐II dye DUT850 (EX/EM = 850/960 nm) by replacing the benzene rings with aliphatic ones.^[^ ^13^ ^]^ Beyond all doubt, the *π*‐elongation strategy effectively generates new NIR‐I/II dyes. The approach often entails complicated rational design and advanced skillful synthesis for experts with a good knowledge of chemistry and physics. In addition, the expansion of the *π*‐conjugation system also gives rise to some undesirable defects of these xanthene dyes, such as large hydrophobic structure, poor water solubility, low fluorescence quantum yields, and serious aggregation‐induced quenching (ACQ) phenomenon.^[^ ^14^ ^]^

An alternative shortcut to yield NIR xanthene is the 10‐position substitution of xanthene scaffold with elements from the carbon, nitrogen, and oxygen groups. As a seminal work, in 2008, Xiao and Qian et al. replaced the oxygen‐bridge atom of rhodamine analogs with silicon to create NIR silicon rhodamine dyes, which was regarded as “a big step for fluorescent dyes in life science” (Scheme 1A).^[^ ^15^ ^]^ Ever since, NIR emissive silicon–rhodamine/fluorescein dyes have been used in fluorescence probe‐based analyzing and sensing of bioactive species and super‐resolution imaging of cellular bio‐events by many researchers.^[^ ^16^ ^]^ Aside from silicon, researchers also discovered that the oxygen‐bridge atom of rhodamine replaced with other group 14 elements, such as germanium (Ge) and stannum (Sn), also produce NIR emissive rhodamines.^[^ ^17^ ^]^ Using the 10‐position atom replacement strategy, phosphinate, phosphine oxide, and sulfone were also introduced to the 10‐position of rhodamine to produce phosphinate‐rhodamine,^[^ ^18^ ^]^ phosphine oxide‐rhodamine,^[^ ^19^ ^]^ and sulfone‐rhodamine^[^ ^20^ ^]^ respectively, whose maximal emission wavelengths exceeded 650 nm. Attentionally, the emission wavelength of Si‐fluorescein/Si‐rhodol is generally much shorter than Si‐rhodamine, located in the red/far‐red (≈600–650 nm) emission ranges.^[^ ^21^ ^]^ The absorption and emission spectra of *X*‐rhodamine (*X* = Si, Ge, Sn, PO_2_, POR, and SO_2_) usually overlay in a great measure due to the small Stokes shifts of ≈20–30 nm. Another challenge for *X*‐rhodamine is the harsh synthetic conditions: the involvement of ultra‐low reaction temperature (−78 °C) and dangerous organic lithium reagents (*n*‐BuLi/*t*‐BuLi).^[^ ^22^ ^]^ Naturally, by combining the two structural modification methods (*π*‐elongation plus 10‐substitution), xanthene dyes can be further optimized and pushed to way longer emission regions, spanning from the NIR‐I range to the NIR‐II range.^[^ ^23^ ^]^ Considering the drawbacks of current methods that fabricated NIR emissive xanthene, a groundbreaking design strategy for NIR xanthene remains highly welcome. Fortunately, the tetrahydroquinoxaline (THQ) modification strategy has emerged as a rising star in constructing NIR emissive xanthene dyes in the recent 5 years (Scheme 1B). In this structural strategy, one amino group of the xanthene scaffold was replaced with a THQ part that sparked tremendous performance promotions in xanthene dyes, such as NIR emissions (>650 nm) and substantial Stokes shifts (≈100 nm).

Against this backdrop, xanthene‐based dyes including rhodamine, fluorescein, and rhodol, in fluorescent imaging have been extensively discussed and reviewed.^[^ ^24^ ^]^ Still, there were no review papers concerning THQ–xanthene dyes so far, to the best of our knowledge. In this review, we focus on elucidating the advent, development, and mechanism of THQ‐modified xanthene (THQ–xanthene) dyes and THQ–xanthene‐based fluorescence probes in sensing, imaging, and theranostic applications. This review would inspire more scientists to craft novel NIR‐I/II xanthene dyes with the THQ modification strategy and could further boost and accelerate the advance of NIR‐I/II THQ‐xanthene in life science research, environmental analysis, and oncological therapy.

## 2 General Design Principles for THQ–Xanthene

**Figure 1:**
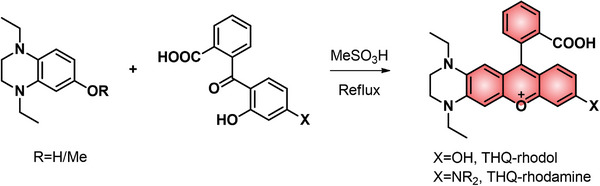
General synthetic route for THQ–xanthene.

In the past 5 years, introducing THQ to xanthene dyes has been substantiated to be an effective and robust method to promote the general visible‐light (<600 nm) emissive dyes to NIR‐I/II emissions, together with large Stokes shifts (usually >100 nm) and considerable brightness. Take THQ–rhodol as an example, the excitation/emission wavelength of rhodol is 535/554 nm, while the excitation/emission wavelength for THQ–Rhodol is 552/653 nm.^[^ ^25^ ^]^ Apart from the extending emission to the NIR‐I range (653 nm cf. 554 nm), the Stokes shift of THQ–rhodol is much larger than that of rhodol (103 nm cf. 19 nm), which can remarkably reduce the overlay of excitation and emission in bioimaging applications and improve signal‐to‐background ratio (SBR). The reasons why the THQ can increase the emission wavelength and Stokes shift can be explained as follows (Scheme 1B). After incorporating THQ into xanthene scaffolds, the asymmetric structures are favorable for increasing the vibronic energy structures of the THQ‐xanthene dyes. Next, through internal conversion, the electron of the excitation state (*S*_1_) transfers from high energy level to low energy vibronic levels and then transfers to the *S*_0_ state. The reduced energy gap (*ΔE*_2_) for THQ–rhodol is much smaller than the energy gap (*ΔE*_1_) of rhodol, resulting in a longer emission and a larger Stokes shift.^[^ ^26^ ^]^ On the other hand, the diethylamine moiety of common rhodol can be facilely twisted and rotated through the C—N bond, leading to the twist‐induced charge transfer (TICT) effect and the decrease of fluorescence emission. However, in the THQ–rhodol, the C—N bond cannot twist or rotate due to the locked diamino group by the piperazine ring.^[^ ^27^ ^]^ In this consideration, the introduction of THQ can unquestionably increase the brightness of the fluorophore to a certain degree. The elongated NIR‐I/II emissions and large Stokes shifts can also be endowed in other fluorophores including rhodol, rhodamine, coumarin, cyanine, and squaraine when these dye scaffolds are reasonably fused with THQ moieties. Like conventional xanthene dyes, sundry THQ–rhodols or THQ–rhodamines can be organically synthesized through the one‐step reaction of tetrahydroquinoxaline derivates and 2‐(2‐hydroxybenzoyl)benzoic acid derivates with good yields under mild conditions (**Figure** **1**). In this review, we use THQ–xanthene dyes, including rhodamine, fluorescein, rhodol, and xanthene‐like scaffolds to elaborate this emerging strategy in developing novel NIR‐I/II dyes and related fluorescent probes, as well as their theranostic applications.

## 3 Evolvement of THQ–Xanthene Based NIR Fluorophores

### 3.1 The Naissance of THQ–Xanthene Fluorophores

**Figure 2:**
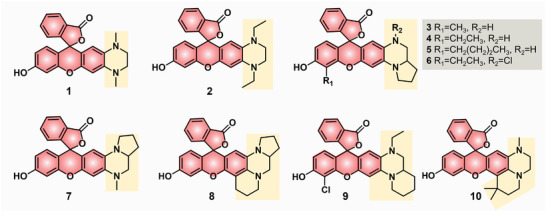
Structures of various THQ–rhodol dyes 1–10.

In 2017, Xian's group first reported an unprecedented class of NIR dyes 1–10 (**Figure** **2**), fusing tetrahydroquinoxaline to rhodol dyes, with favorable NIR emissions (>650 nm) and large Stokes shifts (113–149 nm).^[^ ^28^ ^]^ Originally, they synthesized two new THQ‐fused rhodol (THQ‐rhodol) dyes, 1 and 2, in which the amino group was replaced with the tetrahydroquinoxaline ring. The parent rhodol dyes with two *N*‐alkyl chains generally displayed absorption at ≈520 nm and emission at ≈550 nm, while the THQ‐rhodol dyes 1 and 2 showed absorption peaks at some 540 nm and emissions at 668 nm. From these results, THQ–rhodol dyes displayed NIR emissions (668 nm) and significant Stoke shifts (128 nm). Nonetheless, the fluorescence quantum yields of dyes 1 (0.06) and 2 (0.07) were low in buffer solutions. To boost fluorescence quantum yields, a five‐membered pyrrolidine ring or six‐membered piperidine ring was further linked to the THQ ring at different positions to produce new THQ‐rhodol dyes 3–10. As a result, dyes 3–10 were NIR emissive at 656–706 nm with large Stokes shifts between 111 and 149 nm. Theoretically, the pyrrolidine or piperidine ring could enhance the rigidity of THQ and decrease the twist and rotation of chemical bonds;^[^ ^29^ ^]^ thus, stifling TICT and enhancing fluorescence emission intensities. Interestingly, only the pyrrolidine ring fused on at the *meta*‐position of the endocyclic oxygen atom (dyes 3–6) could significantly improve the fluorescence quantum yields as high as 0.2. For dyes 7–10, the pyrrolidine ring at the *para*‐position and the introduction of chlorine could redshift the emission wavelengths up to 706 nm but have fluorescence quantum yields around 0.06 (**Table** **1**). Overall, the authors first proposed and validated the THQ modification concept that THQ–rhodols have NIR emission wavelengths and large Stokes shift of >110 nm. In addition, some key points to intensify the brightness of the THQ‐fused rhodol dyes by structural modifications have been preliminarily discussed and explored.

**Table 1:** Optical properties of THQ–xanthene dyes 1–33 and their parent dyes

Dyes	*λ*_abs_ [nm]	*ε* [M^−1^⋅cm^−1^]	*λ*_em_ [nm]	Stokes shift [nm]	*Φ*	Brightness [*εΦ*]
Rhodol	≈520	—	≈550	≈30	≈0.15	—
1	539	31 000	668	129	0.06	1860
2	545	30 000	668	123	0.07	2100
3	540	35 500	656	116	0.20	7100
4	543	45 500	656	113	0.21	9555
5	545	32 500	662	117	0.19	6175
6	551	42 000	662	111	0.20	8400
7	539	29 500	676	137	0.06	1770
8	545	30 000	676	131	0.05	1500
9	557	39 000	706	149	0.04	1560
10	542	30 500	666	121	0.06	1830
OP	552	116 000	571	19	0.37	42 920
11	594	153 000	652	58	0.30	45 900
SiP	640	12 100	652	12	0.24	29 040
12	707	—	829	122	—	—
OX	665	111 000	669	4	0.07	7770
13	683	157 000	764	81	0.05	7850
Rh101	564	118 604	587	23	0.91	107 929
14	593	179 000	653	60	0.43	76 970
RhB	553	117 000	572	19	0.53	62 010
15	584	176 000	660	76	0.35	61 600
Rh6G	530	116 000	551	21	0.95	110 200
16	570	142 000	646	76	0.31	44 020
Rh110	498	76 000	520	22	0.88	66 880
17	565	128 000	648	83	0.24	30 720
18	560	71 000	640	80	0.35	24 850
Squaraine	628	222 000	637	9	0.16	35 520
19	680	180 000	755/805	95/125	0.068	12 240
Cy3	547	173 000	562	15	0.12	20 760
20	692	247 000	752	60	0.23	56 810
Cy5	641	137 000	660	19	0.20	27 400
21	780	273 000	836	56	0.027	7371
22	552	—	653	101	—	—
23	565	—	680	115	—	—
24	564	—	698	134	0.064	—
25	574	52 200	673	99	0.10	5220
26	571	66 000	661	90	0.09	5940
27	576	70 000	649	73	0.32	22 400
28	575	70 200	637	62	0.59	41 400
29	575	82 500	636	61	0.51	42 100
30	578	89 700	634	56	0.74	66 400
31	557	88 800	590	33	0.44	39 100
RhB	553	1 050 000	580	27	0.31	32 500
32a	910	—	979	69	0.0139^a)^/0.0035	2933
32b	941	—	996	53	0.0122^a)^/0.0026	2330
32c	860	—	922/999	73/133	0.023^a)^/0.013	4710
32d	856	—	929/989	62/139	0.0142^a)^/0.0115	2812
O‐HD	692	79 000	710	18	0.30	23 700
33a	806	69 600	942	136	0.001	6960
33b	802	49 000	886/943	84/141	0.0017	8330
33c	837	79 200	886/944	49/107	0.0031	24 552
33d	854	103 200	895/936	41/82	0.0028	28 896

^a)^ Measured in dichloromethane.

### 3.2 Generalizing THQ Modifications to Common Fluorophores

**Figure 3:**
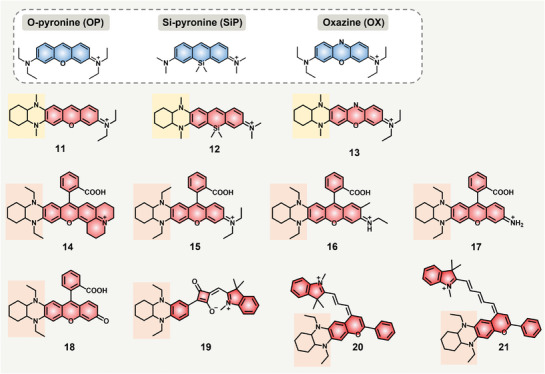
Conventional dye‐based DQ‐modified NIR fluorophores 11–21.

In 2018, Yuan and Zhang's group developed 11 different fluorophores 11–21 by the THQ modification strategy of appending 1,4‐diethyl‐decahydro‐quinoxaline (DQ) moiety, a structure similar to THQ, to common symmetric fluorophores, such as fluorescein, rhodamine, oxazine, squaraine, and cyanine dyes (**Figure** **3**).^[^ ^30^ ^]^ These new asymmetric DQ‐modified fluorophores have larger Stokes shifts, longer emission wavelengths, and better photostability than their parent fluorophores. Generally, many efforts have been dedicated to developing novel fluorophores with longer NIR emissions, relying on an established theoretical consensus that reducing the energy gap of HOMO and LUMO can remarkably red‐shift the emission to NIR‐I/NIR‐II emission ranges.^[^ ^31^ ^]^ In sharp contrast, less attention is paid to how to enlarge the Stokes shift. Recognizing this predicament, the authors conjectured that grafting asymmetric electronic structures to conventional dyes can enhance the vibronic contributions of HOMO and LUMO and resultant internal conversion. Then the electrons at the excited states transit to lower vibronic levels, leading to augmentation of Stokes shifts. As a proof of concept, three simple fluorophore scaffolds including O‐pyronine (OP), Si‐pyronine (SiP), and oxazine (OX) were introduced with DQ structures to produce new fluorophores 11–13, accordingly. The Stokes shifts for OP, SiP, and OX were 19, 12, and 14 nm, respectively, and the DQ‐modified fluorophores 11–13 exhibited Stokes shifts of 58 nm (11), 122 nm (12), and 81 nm (13). Moreover, all the emissions of the DQ‐modified fluorophores promoted much longer NIR emission ranges, 651 nm for 11, 840 nm for 12, and 780 nm for 13, compared with the short emissive parent fluorophores. Along with the excellent properties, their absorption coefficients and brightness were all increased. Notably, the DFT calculated HOMOs and LUMOs results also confirmed the asymmetric vibronic features of fluorophores 11–13. To further prove that the DQ modification can be a general method to increase the Stokes shifts of fluorophores, rhodamine dyes (rhodamine 101, rhodamine B, rhodamine 6G, and rhodamine 110) were incorporated with DQ moieties to produce new dyes 14–17. DQ‐modification rhodamines have larger Stokes shifts in the range of 60–83 nm (60 nm for dye 14, 76 nm for dye 15, 76 nm for dye 16, 83 nm for dye 17), which are threefold larger than the parent fluorophores, usually ≈20 nm. Except for the increase of Stokes shift, the DQ ring modification can also push the emissions into a far‐red emission range (*λ*_em_ = 646–660 nm), which is at least 70 nm redshift than the original rhodamines. At the same time, the absorption was significant in a bathochromic way, while the fluorescence quantum yields dropped obviously but resulted in equal brightness with parent rhodamines across‐the‐board. To prove the universality of this design principle, the authors continued to incorporate DQ into rhodols, squaraine, and cyanine dyes to produce novel NIR fluorophores 18–21. After the DQ ring expansion, the DQ‐rhodol dye 18 had a threefold increase of Stokes shift, from 26 to 80 nm. The Stokes shifts of squaraine increased 13‐fold, from 9 to 125 nm. After the indoline in Cy3/cy5 was replaced with benzoquinoxaline, the Stokes shifts increased from 15 to 60 nm for Cy3 and from 19 to 56 nm for Cy5, severally. More importantly, the emission wavelengths of dyes 18–21 were remarkably redshifted by 100–170 nm and located in the 640–836 nm NIR range (Table 1). Of note, DQ was a structurally updated version of THQ, in which the THQ moiety was further restricted by the cyclohexane ring and improved the fluorescence quantum yields of DQ–xanthene dyes. This work confirmed that the DQ ring modification on common dyes can be an effective and universal structural tactic to develop NIR fluorophores.

### 3.3 Halogen's Effect on THQ–Rhodol Fluorophores

**Figure 4:**
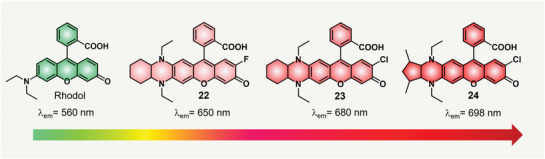
Prolonging the emission of THQ–rhodol with halogen's substitution and tunable rigidity.

In 2022, Liu and Kim et al. also studied the halogen's effects on the emissions of THQ–rhodol dyes 22–24 (**Figure** **4**).^[^ ^32^ ^]^ Compared with dye 18, the fluorine‐substituted THQ–rhodol dye 22 showed a small redshift of 10 nm in the maximal fluorescence emission and a Stokes shift of 101 nm. Further, they created a chlorine‐substituted THQ–rhodol dye 23 and found that chlorine can enhance a larger redshift of 30 nm in emission when compared to dye 22. To further enhance the fluorescence performances of dye 22, the other side of the THQ moiety was locked with a five‐membered cyclopentane ring to enhance the rigid structure and planarity to produce THQ–rhodol dye 24. With these modifications, the emission band of dye 24 peaked at 698 nm and the Stokes shift was 134 nm. These results demonstrated that the halogen substitution on the phenol part of the rhodol scaffold can effectively enhance the emission wavelengths. Although the emission wavelength of THQ–rhodol can be advanced in the NIR range with these modifications, the fluorescence quantum yield of these rhodol dyes dropped significantly. For instance, the fluorescence quantum yield of dye 24 in PBS was 6.4%.

### 3.4 Systematic Improving Performance of THQ–Xanthene Fluorophores

**Figure 5:**
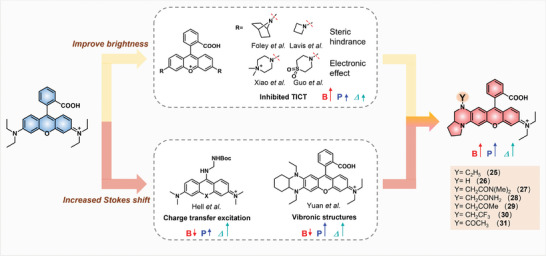
A general structural strategy to improve the photostability, brightness, and Stokes shift of THQ–rhodamine dyes 24–30. *B* indicates brightness, *P* indicates photostability, and *Δ* indicates Stokes shift.

Since the noteworthy Stokes shift and photostability of THQ–xanthene dyes, researchers strived to use these xanthene dyes in stimulated emission depletion (STED)‐based super‐resolution imaging.^[^ ^33^ ^]^ To this end, in 2022, Yuan and Wang et al. reported a synergistic strategy to design THQ–rhodamines (25–31) with good photostability, high brightness, and large Stoke shifts together via the inhibition of TICT and enhancement of vibronic structures (**Figure** **5**).^[^ ^34^ ^]^ In general, the fluorescence of the rhodamine is easily quenched due to the formation of TICT in an excited state, in which the donor part and fluorophore core are at ≈90° angle. To address this issue, introducing an azetidine or aziridine ring^[^ ^35^ ^]^ to replace the dialkylamino group can greatly enhance the brightness of rhodamine via the steric hindrance to suppress the TICT. Further studies revealed that replacing the dimethylamino group with a quaternary piperazine/sulfone‐fused piperidine group^[^ ^36^ ^]^ can also decrease the strength of donor and acceptor interactions, augmenting the energy barrier to enter the TICT state and giving rise to high fluorescence quantum yields. In brief, steric hindrance and electronic push–pull effect are the two paramount considerations to impede TICT and enhance the brightness of dyes. Moreover, grafting THQ to rhodamine fluorophore was confirmed as an effective way to enlarge the Stokes shift and photostability via increasing the vibronic structure. Enlightened by this knowledge, the authors synthesized a series of octahydropyrrolo[1,2‐a] pyrazine‐fused rhodamines 25–31 in which the pyrazine was substituted at the amino group with different electron‐donating/electron‐withdrawing groups. Except for dye 31 with *N*‐acetyl octahydropyrrolo[1,2‐a] pyrazine moiety, other dyes showed at least a two to threefold increase in Stokes shift, varying from 56–99 nm (Table 1). In addition, the dyes with electron‐accepting *N*‐substituted groups showcased significant increases in fluorescence quantum yields and brightness. Among them, dye 30 with 2‐(2,2,2‐trifluoroethyl) octahydropyrrolo[1,2‐a] pyrazine moiety displayed a moderate Stokes shift of 56 nm and the highest fluorescence quantum yield of 0.74, as well as the largest brightness of 66 400 m ^−1^⋅cm^−1^. This work proposed and confirmed a synergistic strategy by tuning steric hindrance and electronic push–pull effect to obtain an ideal dye used for super‐resolution imaging with the improvement and balance of photostability, brightness, and Stokes shift.

### 3.5 Evolution of THQ–Xanthene‐Based NIR‐II Fluorophores

**Figure 6:**
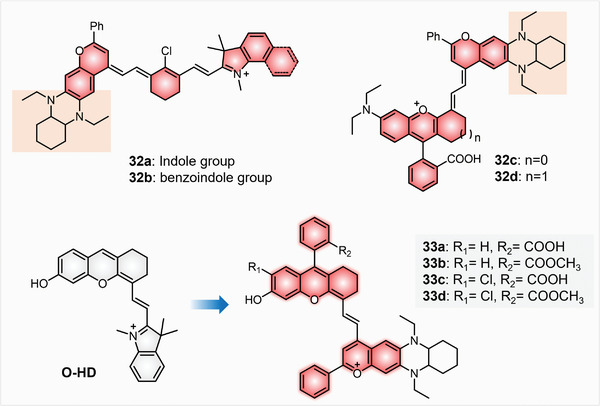
NIR‐II emissive DQ‐xanthene dyes 31–33.

The current molecular NIR‐II dyes mainly rely on two fluorophore types, one is donor–acceptor–donor (D–A–D) benzobisthiadiazole (BBD) fluorophores, and another is extended *π*‐conjugated cyanine dyes.^[^ ^37^ ^]^ Howbeit, the D–A–D BBD fluorophores have small absorption coefficients (<10^4^ m ^−1^⋅cm^−1^), chemical instability in the air, and sensitivity to basic or reducing reaction conditions.^[^ ^38^ ^]^ By contrast, cyanine dyes can be easily evolved into NIR‐II dyes by extending the polymethine chain and enhancing the strength of donors and acceptors. Beautiful yet incomplete, these cyanine‐based NIR‐II dyes define by poor photo/chemo‐stability, small Stokes shift, and solvatochromic quenching for a long time.^[^ ^39^ ^]^ These drawbacks may be ascribed to the strong *π*–*π* stacking interaction between the dyes and symmetrical electronic structures with less vibrational structures.^[^ ^40^ ^]^ In this situation, in 2021, Zhang and collaborators brought about that increasing the steric hindrance or reducing the *π*‐system of the end‐groups could attenuate *π*–*π* interaction to restrain the solvation quenching of cyanine dyes.^[^ ^41^ ^]^ Introducing two different donor moieties with large hindrances to increasing the ICT and asymmetry of cyanines would enhance the photo/chemo‐stability and amplify the Stokes shifts. Ergo, one 1,4‐diethyl‐decahydro‐quinoxaline (DQ) benzopyran group was introduced to the classic cyanine scaffold to produce new NIR‐II dyes 32a and 32b. To easily develop activatable NIR‐II probes, rhodamine moiety was hybridized with the DQ benzopyran group through polymethine skeleton to afford NIR‐II dye 32c and 32d (**Figure** **6**). All four dyes displayed absorption peaks in 850–950 nm, and the emission wavelengths were centered at 979, 996, 999, and 989 nm, respectively. The Stokes shifts were 69, 53, 133, and 139 nm, larger than previous NIR‐II cyanine dyes. Markedly, the brightness of the prepared dyes was 3.6‐ (32d) to 10‐fold (32a) stronger than ICG, a standard and commercial NIR‐II dye. Taking advantage of the unique spirocyclization and spirolactam open chemistry of rhodamine lactam structures, dyes 32c and 32d can be easily devised target (pH, ATP, Hg^2+^)‐responsive NIR‐II fluorescent probes. The recognition sites based on spirocyclization and spirolactam‐open chemistry of rhodamine are limited for some analytes including metal ions and ATP. In contrast, activatable probes based on the caged/decaged reactions of hydroxyl or amino groups can be applied for detecting a much wider range of species, such as metal ions, enzymes, and RONS. In 2022, Yuan's group developed a series of hydroxy‐containing hemicyanine (O‐HD)‐based dyes 33a–33d with fluorescence tunable sites for NIR‐II imaging (Figure 6).^[^ ^42^ ^]^ Based on the O‐HD scaffold, DQ benzopyran moiety replaced the indole moiety of O‐HD for enhancing the donor strength and Stokes shifts. Further, grafting halogen to the *ortho*‐position of the phenol group to reduce pK_a_ promoted the response performance of its molecular probes. Meanwhile, the graft of 2‐benzoic acid/2‐benzoic acid ester to O‐HD scaffold can increase the chemical stability by preventing nucleophilic attack of the 9‐position of the xanthene ring. All the absorption wavelengths were located at 800–860 nm, and all the emission wavelengths exceeded 900 nm, reaching to the NIR‐II emission range. The Stokes shift varied from 82 to 148 nm, 4.5 to 8.2‐fold than their parent dye O‐HD (Table 1). Dyes 33c and 33d with chlorine substitution presented lower pK_a_ values of 6.3 and 6.5, compared to 33b (pK_a_ = 8.0). As evidenced by these strides, we are confident that the THQ modification strategy is promising to create new NIR‐II emissive xanthene dyes through minor structural changes for NIR‐II in vivo imaging.

## 4 Biological Applications of THQ–Xanthene Dyes

### 4.1 THQ–Xanthene‐Based Fluorescent Probes

The large Stokes shifts and NIR emissions, as well as the facile preparation, make THQ–xanthene dyes resolve the shortcomings of conventional xanthene dyes and enjoy unparalleled superiorities in disease/cancer‐related imaging, such as high SBR readout signals, accurate results with high sensitivity. Therefore, a panel of THQ–xanthene‐based NIR‐I/II fluorescent probes for small molecules, reactive oxygen, and nitrogen species (RONS), enzymes, and metal ions in biological processes has been developed in recent years.

#### 4.1.1 Probe for Small Molecules

**Figure 7:**
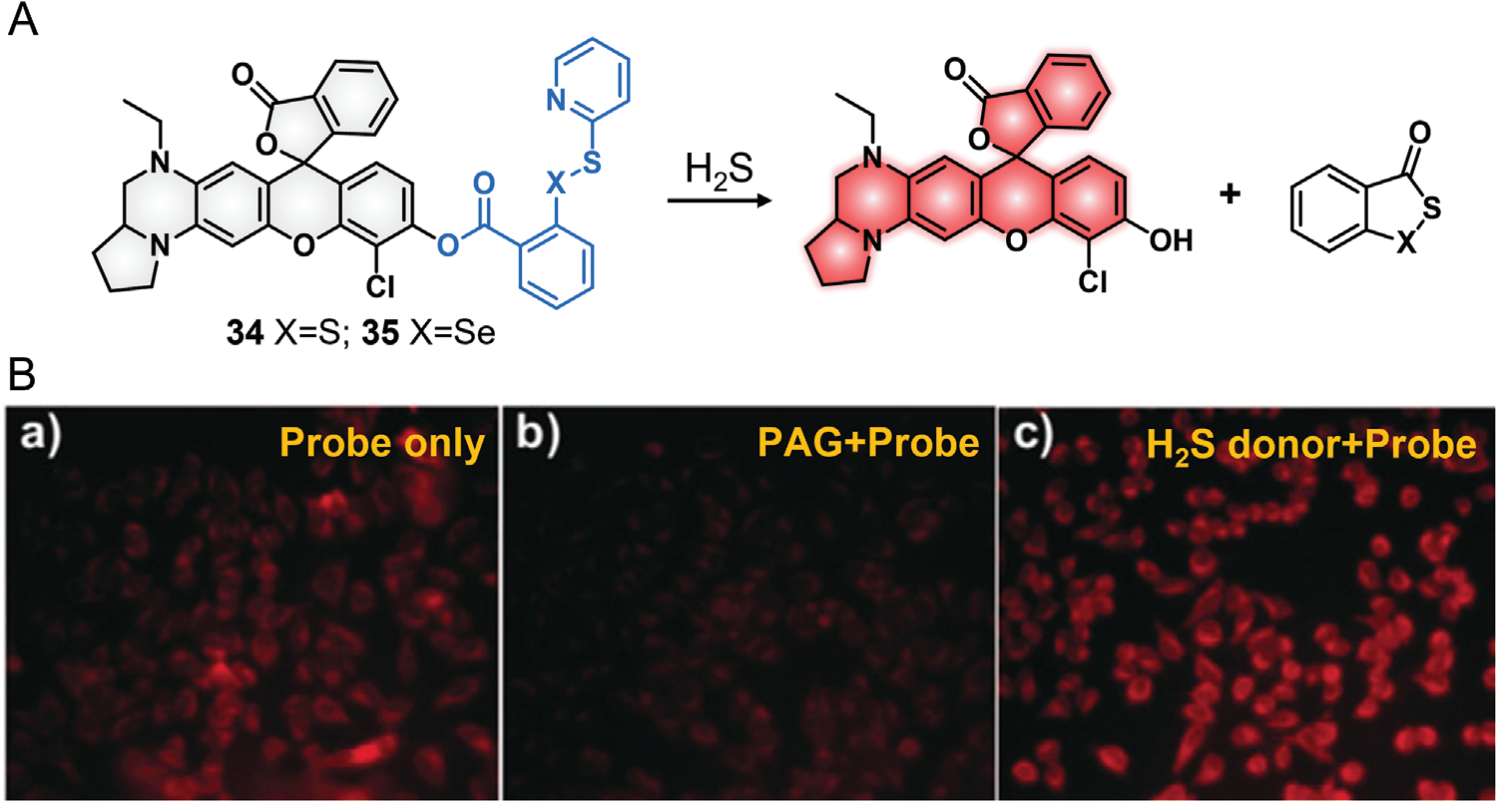
H_2_S‐responsive probes 34 and 35. A) Reaction principle for H_2_S‐activated fluorescent probes 34 and 35. B) Imaging cellular H_2_S with probe 35. Reproduced with permission_._^[^ ^28^ ^]^ Copyright 2017, Wiley‐VCH.

**Figure 8:**
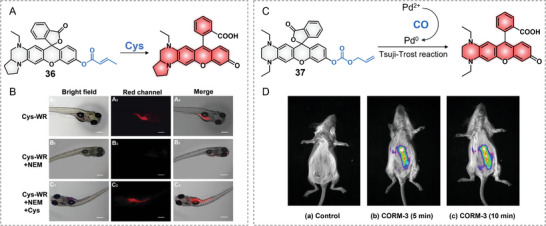
Probes for Cys and CO. A) Chemical structure of probe 36. B) Detecting Cys in zebrafish. Reproduced with permission.^[^ ^44^ ^]^ Copyright 2019, Elsevier, B.V. C) Chemical structure of probe 37. D) CO detection in mice with probe 37. Reproduced with permission.^[^ ^45^ ^]^ Copyright 2020, Elsevier, B.V.

In 2017, Xian's group took the THQ‐modified rhodol to develop two probes for detecting intracellular H_2_S (**Figure** **7**A).^[^ ^28^ ^]^ THQ–rhodol was linked to H_2_S‐sensitive chemical moieties that contain —S—S— or —Se—S— to produce probes 34 and 35, separately. The disulfide bond (—S—S—) has long been used for sensing H_2_S in fluorescence probe design; however, concerns still exist that the disulfide bond also can be cleaved by biological thiols; thus, lowering the sensing properties of disulfide‐involved probes.^[^ ^43^ ^]^ In general, the Se—S bond can react to H_2_S but not with thiols. Probe 34 containing the S—S bond displayed a significant fluorescence increase to H_2_S and other biothiols (GSH, Cys, and Hcy), while probe 35 containing the Se—S bond only showed a fluorescence increase to H_2_S over amino acids and biothiols. Then, probe 35 was selected to image intracellular H_2_S. The H_2_S biosynthesis inhibitor (propargylglycine, PAG)‐induced reduced fluorescence and the H_2_S donor (*N*‐(benzoylthio)benzamide)‐induced increased fluorescence proved the feasibility of using the probe to detect endogenous H_2_S in cells (Figure 7B). By the same fluorophore of 34 and 35, a cysteine‐activatable fluorescent NIR probe was also exploited in 2019 by Yu et al. and used to detect Cys changes in cells and zebrafish (**Figure** **8**A,B).^[^ ^44^ ^]^

In 2020, Feng's group developed a NIR emissive fluorescence probe 37 for in vivo surveilling carbon monoxide (CO) at the animal level (Figure 8C).^[^ ^45^ ^]^ Probe 37 is comprised of a THQ–rhodol fluorophore and an allyl carbonate group as a CO‐specific reaction trigger and fluorescence quencher. In the ternary component detection system, CO can reduce palladium ion (Pd^2+^) to Pd (0), and Pd (0) can quickly break the carbonate bond to release the fluorophore by the Tsuji–Trost reaction and eventually restore the fluorescence emission. The probe responded to 0–10 µm CORM‐3 in 10 min with a 28‐fold fluorescence increase at 676 nm. The large Stokes shift (*Δλ* = 135 nm), good water‐solubility, and NIR emission (≈680 nm) enabled the sensitive surveillance of CO release from CORM‐3 in cells, zebrafish, and living mice (Figure 8D).

**Figure 9:**
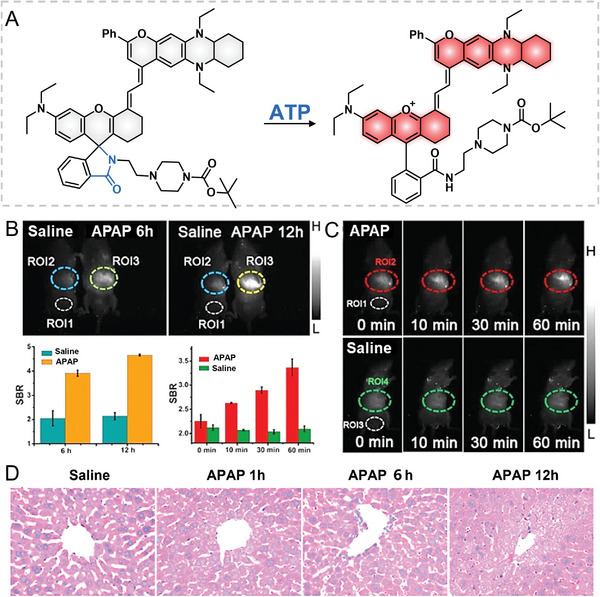
ATP‐responsive NIR‐II probe 38. A) Chemical structure of 38. B) NIR‐II imaging of APAP's hepatoxicity. C) Real‐time imaging of APAP's hepatoxicity. D) H&E study of APAP's hepatoxicity. Reproduced with permission.^[^ ^41^ ^]^ Copyright 2021, Wiley‐VCH.

In 2021, Yuan et al. developed an ATP‐activatable NIR‐II probe 38 for imaging drug's hepatoxicity in vivo (**Figure** **9**A).^[^ ^41^ ^]^ Probe 38 containing a rhodamine spirolactom showed no fluorescence emission due to the destruction of the *π*‐conjugate system. After reacting with ATP, the spirolactom ring was open and the large *π*‐conjugate system was restored, resulting in a surge of the fluorescence emission at 918 nm. A good selectivity of probe 38 to ATP was confirmed by comparing its responses to biological species including ADP, AMP, GTP, biothiols, and ATP. Given that ATP is a critical signal molecule in damaged and stressed cells, probe 38 was further harnessed to estimate the drug‐induced hepatoxicity through ATP fluorescence monitoring. The results denoted a significant NIR‐II fluorescence increase in acetaminophen (APAP)‐induced hepatoxicity of mice, and the fluorescence was also incrementally enhanced with the prolonged APAP treatment time. The APAP‐induced hepatoxicity in living mice was real‐time imaged in 0–1 h (Figure 9B,C) and corroborated by H&E staining (Figure 9D). Over and above the APAP‐induced hepatoxicity, the CCl_4_‐caused hepatoxicity of mice could be imaged through in vivo NIR‐II imaging.

#### 4.1.2 Probes for RONS

**Figure 10:**
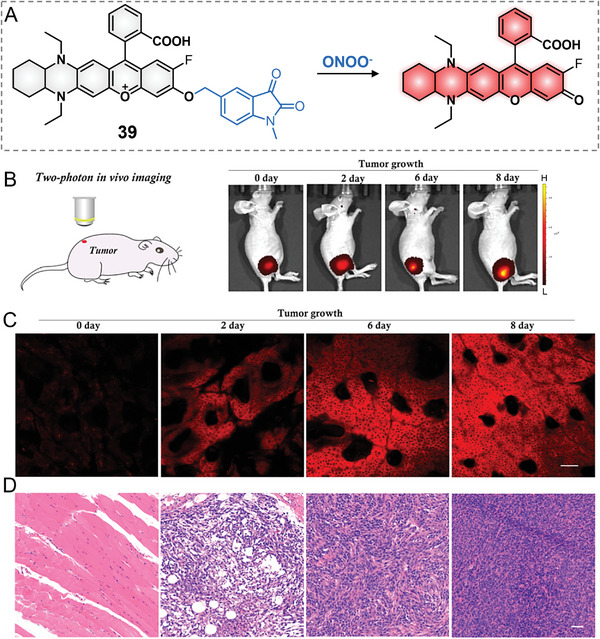
One‐photon and two‐photon imaging of tumorous ONOO^−^ with probe 39. A) Reaction principle for probe 39 and ONOO^−^. B) Two‐photon microscopic imaging ONOO^−^ in tumors. C) One‐photon in vivo imaging ONOO^−^ in tumors. D) H&E results of normal and tumor tissues of mice. Reproduced with permission.^[^ ^25b^ ^]^ Copyright 2020, American Chemical Society.

In 2020, Mao et al. reported a THQ‐modified rhodol‐based two‐photon excitable probe 39 for the selective imaging of ONOO^−^ in the onset and development of tumor progress (**Figure** **10**).^[^ ^25b^ ^]^ In this probe, a fluorine‐substituted THQ‐modified rhodol was selected as two‐photon excitable NIR fluorophore for the first time, and 1‐methylindoline‐2,3‐dione derivative was selected as an ONOO^−^ specific recognition group and a fluorescent quencher. Probe 39 emitted a 17‐fold fluorescence increase at 653 nm in response to 0–35 µm ONOO^−^. In addition, the fluorescence intensity for the probe and ONOO^−^ could be leveled off at 240 s. Notably, the probe also displayed excellent selectivity to ONOO^−^ over other metal ions and RONS. Further, probe 39 also demonstrated a considerable active two‐photon cross‐section of 170 GM at 820 nm, supporting the superb feasibility of two‐photon NIR‐to‐NIR (Ex/Em 820/653 nm) imaging of biological processes with probe 39.^[^ ^46^ ^]^ The probe reported the fluctuations of endogenous and exogenous ONOO^−^ by two‐photon imaging. Excitedly, the probe was utilized for the one‐photon and two‐photon imaging of ONOO^−^ in tumor development processes in vivo. The one‐photon in vivo imaging clearly revealed the increased ONOO^−^ production along with the tumor development on different days (0, 2, 6, and 8 days). By contrast, two‐photon in vivo imaging provided much higher resolution images at the cellular level, and even the tumor on day 2 displayed a much more significant fluorescence increase (11.5‐fold vs 1.5‐fold) under two‐photon imaging than that of one‐photon in vivo imaging. By the marriage of one‐photon and two‐photon imaging with probe 39, researchers would have a versatile imaging method for studying ONOO^−^ influxes in tumors both at a whole animal and a local regional level. Of particular interest, this work afforded the specific study of the two‐photon absorption performance of THQ–rhodol in solution and in vivo for the first time.

**Figure 11:**
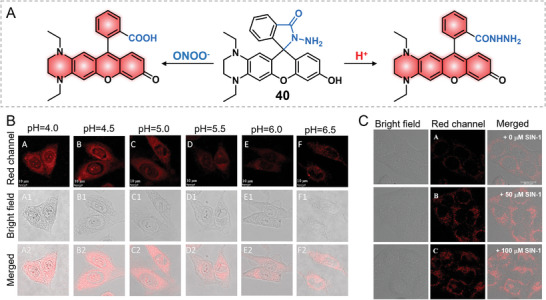
ONOO^−^ and pH dual‐activated probe 40. A) Chemical structure of probe 40. B) Imaging cellular pH. C) Microscopic detection of exogenous ONOO^−^. Reproduced with permission^.[^ ^49^ ^]^ Copyright 2021, Elsevier, B.V.

Peroxynitrite and lysosomes play key roles in living systems. Generally, the pH values of lysosomes are maintained at 4.0–5.5, which is important to secure the normal metabolic functions of lysosomes.^[^ ^47^ ^]^ Studies showed that the increasing cellular ONOO^−^ can result in lysosomal membrane permeabilization (LMP) and cell death.^[^ ^48^ ^]^ Thus, new methods to research the connections between lysosomal ONOO^−^ and lysosomal pH are a necessity. In 2021, Feng's group developed a THQ‐rhodol‐based activatable probe 40 for the dual‐detection of lysosomal ONOO^−^ and pH **(Figure 11)**.^[^ ^49^ ^]^ The benzoyloxy group of THQ–rhodol reacted with hydrazine to yield probe 40. Upon the addition of ONOO^−^ or H^+^, the rhodol lactam ring was broken and the fluorescence was remarkedly increased. Probe 40 displayed rapid fluorescence responses at 686 nm to acidic pH and a fluorescence increase at 678 nm for ONOO^−^. Multiple amino groups probably contributed to the lysosome accumulation property of the probe. Then, the lysosome‐targeting ability of 40 was further corroborated by the co‐localization imaging with Lyso‐Tracker green with a Pearson overlay coefficient of 0.87. The changes of intracellular pH or ONOO^−^ at different conditions, such as exogenous pH or ONOO^−^, endogenous pH during cellular heatstroke, could be sensitively monitored with the probe. Frankly, there may be a fly in the ointment for this work; the cellular fluorescence changes in complex conditions would be confusing due to the almost identical emissions of the probe to pH and ONOO^−^ (Figure 11).

**Figure 12:**
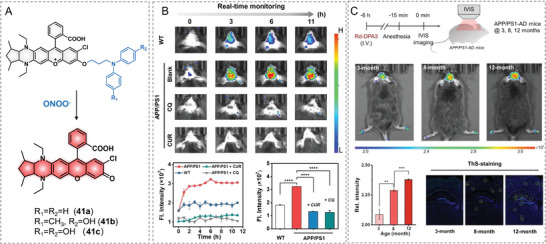
Activity‐based probes for ONOO^−^ in AD. A) Reaction for probe 41a–c and ONOO^−^. B) Real‐time imaging ONOO^−^ in AD mouse brains. C) Evaluation of the severity of AD by measuring ONOO^−^. Reproduced with permission.^[^ ^32^ ^]^ Copyright 2022, Wiley‐CH.

In vivo imaging‐based diagnosis of Alzheimer's disease (AD) has garnered a broad spectrum of interest for a long time but the progress has not met the requirements for the early diagnosis and therapy for AD.^[^ ^50^ ^]^ The dilemma may be a result of various reasons, such as poor brain accumulation of probes caused by the blood–brain barrier (BBB) and disputed interdependency of biomarkers (amyloid *β*‐protein, tau‐protein) and AD.^[^ ^51^ ^]^ In 2022, Kim and Liu et al. reported an ONOO^−^‐activatable NIR fluorescent probe for studying the intricate roles of ONOO^−^ in AD, which aims to promote the early diagnosis of AD and understanding of AD pathogenic mechanism (**Figure** **12**A).^[^ ^32^ ^]^ The newly designed THQ–rhodol, featuring a NIR emission, a large Stokes shift, a small molecular weight, and tunable lipophilicity, endowed the possibility of using the rhodol scaffold to cross BBB and enrich in the brain parenchyma according to the Rule of five (RO5)^[^ ^52^ ^]^ for brain‐targeted drugs. On molecular design, the authors introduced 1,3‐dimethylcyclopentane to the THQ ring to enhance the rigidity and chlorine to the vicinal position of the phenol part to get a new THQ–rhodol with a maximal emission at 700 nm. In addition, *p*‐aminophenol moiety was prone to be oxidized by several RONS, such as ONOO^−^, ClO^−^, and ⋅OH. We expected that *p*‐aminophenol modified with suitable substitution groups could meliorate the selectivity to ONOO^−^. Thus, the fluorophore and *p*‐aminophenol derivates are linked by triple methylene (—CH_2_CH_2_CH_2_—) to form probe 41a–c. After structural optimization, probe 41c can respond to 0–10 µm ONOO^−^ with a 50‐fold fluorescence increase at 698 nm, supportive of the excellent sensitivity of the probe toward ONOO^−^. Of note, probe 41c reacted with ONOO^−^ especially over other ROS, even the concentrations of other ROS as high as 0.3 mm. In addition, probe 41c showed little fluorescence changes to proteins (BSA, HSA, A*β* oligomer) and was kept stable in cell culture media for 12 h, such as PBS, FBS–PBS, PRMI 1640, and DMEM. The in vitro BBB penetration rate was calculated as 55.5% by the transwell method, supporting the probe that can cross BBB and in vivo imaging of the AD brain. The ONOO^−^ imaging ability of 41c was solidly confirmed by imaging cellular ONOO^−^. 41c was first used in the in vivo imaging of AD mouse brains and reflected the elevated ONOO^−^ production in AD brains than the control mouse brains (Figure 12B). Further, sparse fluorescence was observed in AD mouse brains when the AD mice were pretreated with anti‐AD chemicals, such as chloroquin (CQ) and curcumin (CUR). The results established that the administration of CQ and CUR in AD treatments was closely engaged with regulating RONS levels. Furthermore, AD mice at different phases were in vivo imaged with the probe, and the imaging results disclosed a positive correction between ONOO^−^ level and the development of AD (3‐, 8‐, and 12‐month) (Figure 12C). In conclusion, this research preliminarily pictured the connections between AD and ONOO^−^, and in vivo detecting ONOO^−^ in AD brains with probe 41c can work as an auxiliary method for early diagnosis of AD.

**Figure 13:**
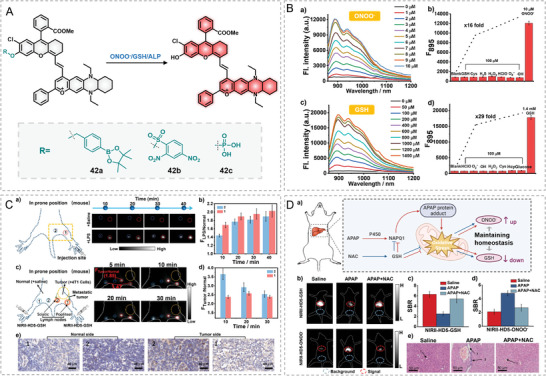
A general platform for designing activatable NIR‐II probes. A) Chemical structures of probe 42a–c. B) Fluorescence responses of 42a/42b to ONOO^−^/GSH. C) NIR‐II imaging of LPS‐treated lymph nodes with 42b. D) Dual‐imaging of ONOO^−^ and GSH fluxes in drug‐caused hepatotoxicity with 42a and 42b, respectively. Reproduced with permission.^[^ ^42^ ^]^ Copyright 2022, Wiley–VCH.

In 2022, Yuan's group developed a universal NIR‐II fluorophore platform for fabricating various target‐activatable probes by caging or uncaging the hydroxyl group with different target recognition groups, such as the benzeneboronic acid pinacol ester derivative for ONOO^−^ (42a), the 2,4‐dinitrobenzenesulfonyl group (42b) for GSH, and the phosphate group for ALP (42c) (**Figure** **13**A).^[^ ^42^ ^]^ Probe 42a responded to ONOO^−^ with an “off–on” fluorescence increase (*F/F_0_* = 16) at 895/936 nm under the excitation of 800 nm. Similarly, probe 42b displayed a “turn‐on” fluorescence (*F/F_0_* = 29) at 895/936 nm to 0–1.4 mm GSH. Both 42a and 42b displayed fabulous sensing selectivity for specific targets (ONOO^−^/GSH) over other biological species (Figure 13B). 42a was first harnessed in in vivo NIR‐II imaging of ONOO^−^ in the lipopolysaccharide (LPS)‐induced lymphatic inflammation (Figure 13C). In this mouse model, both popliteal and sciatic lymph nodes presented a build‐up of in vivo NIR‐II fluorescence signal post‐administration of 42a, and the popliteal lymph nodes exhibited a twofold fluorescence increase at 30 min. In tumor metastasis, tumor cells often travel from primary tumor sites to other organs through lymphatic vessels. In addition, the GSH level in tumor cells is more highly expressed (usually at 2–10 mm) than in normal cells. In this respect, a GSH‐responsive NIR‐II probe might indicate the migration process of tumor cells through lymphatic vessels by the GSH‐triggered fluorescence. To verify this hypothesis, a 4T1 tumor was grown on the buttock of a mouse, and then probe 42b was intradermally injected. The NIR‐II fluorescence signal of the popliteal lymph nodes near the tumor side of mice exhibited remarked increase than that on the other side of mice, proving the migration of tumor cells in lymphatic vessels. It is well‐established that cellular GSH is depleted while the production of ONOO^−^ is augmented in the APAP‐induced hepatoxicity process.^[^ ^53^ ^]^ To further confirm the in vivo diagnostic ability of these probes, the authors further used 42a and 42b to respectively visualize the dynamic changes of ONOO^−^ and GSH in APAP‐induced liver injury for the first time (Figure 13D). In addition, NIR‐II imaging also illustrated that NAC ameliorated APAP's toxicity to livers by restoring GSH levels and inhibiting the burst of ONOO^−^ in livers. The interrelated changes of ONOO^−^ and GSH in mouse livers vindicated the potential of these probes for in vivo diagnosis of liver injury‐related diseases. Regardless of the success, it also needs to point out that the identical fluorescence responses of these probes to the two analytes make it impossible to simultaneously visualize the GSH and ONOO^−^ in one mouse, which should be well addressed in future work.

**Figure 14:**
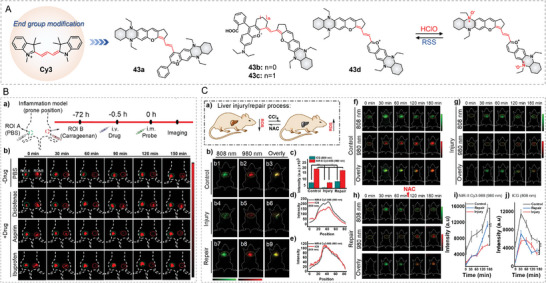
THQ–xanthene‐based reversible probes for oxidative stress. A) End group modification‐based design of 43a–d. B) Evaluation of anti‐inflammation efficiency of NAISDs (diclofenac, aspirin, and ibuprofen) in inflammation mice. C) CCl_4_‐caused liver injury and its dynamic repair with NAC. Reproduced with permission.^[^ ^54^ ^]^ Copyright 2022, Wiley‐VCH.

Given that biological substances constantly keep dynamic changes in living bodies, most previously reported activatable NIR‐II probes without reversible response performance are unable to trace the dynamic changes of cellular events. For instance, the cell redox cycle is generally maintained by the levels of RONS (O_2_^•−^, H_2_O_2_, HClO, ONOO^−^, etc.) and biological reductants (GSH, Hcy, H2S, reductase, etc.). In 2022, Yuan and Ren developed a NIR‐II probe 43d that represented reversible responses to HClO and H_2_S in vivo (**Figure** **14**A).^[^ ^54^ ^]^ The absorption/emissions of trimethylcyanine (Cy3) dyes can be easily redshifted to the NIR range by extending the vinylene number of the Cy3 scaffold. Empirically, one vinylene results in ≈100 nm bathochromic shift in the absorption and emission wavelengths.^[^ ^55^ ^]^ Howbeit, the vinylene‐extended NIR‐emissive cyanine dyes have poor chemo‐stability and photostability. Through end group modification strategy, THQ‐decorated benzopyran and its derivatives replaced indoline end‐groups of the Cy3 scaffold to obtain new NIR‐II dyes 43a–d. As a result, all the absorption and emissions of 43a–d were located at the NIR‐II ranges (938/1001 nm, 956/1005 nm, 962/1015 nm, and 988/1058 nm). These dyes exhibited better tolerance with ROS and RSS than commercial NIR‐II dyes, such as ICG, IR 26, and IR1048. Meanwhile, 43a–d could be oxidized by HClO confirmed by the absorption changes and HRMS results. The HRMS supported that the quinoxaline group in 43a–d could react with HClO to form *N*‐oxide products; thus, causing absorption and fluorescence decreases. In addition, RSS like H_2_S, GSH, Hcy, and Cys could reduce the *N*‐oxide groups to amino groups, restoring the NIR‐II absorption and fluorescence. Due to the longest absorption and emission wavelengths (988/1058 nm), probe 43d was preferred as the proof‐of‐concept reversible probe for oxidative stress. The absorption at 980 nm and emission at 1040 nm for 43d was dropped with 0–15 µm HClO, while the intensities of absorption at 980 nm and emission at 1040 nm were restored upon adding 0–3.5 µm H_2_S. Impressively, the reversible response to HClO and H_2_S could be cycled at least four times, suggestive of the probe's excellent reversible redox response ability. Of note, H_2_S displayed a much more efficient reduction of the *N*‐oxide to an amino group than GSH, Cys, and Hcy. Then, probe 43d was used to estimate drug efficacy in a carrageenan‐induced acute inflammation mouse model (Figure 14B). The NIR‐II fluorescence signals of the inflamed site decreased gradually, while the signal of the control site (PBS only) almost kept unchanged. When the inflamed sites were further treated with anti‐inflammation drugs, NAC, the restoration of NIR‐II signals could be observed. These results confirmed the dynamic HClO generation in the inflammation and anti‐inflammation processes. The author further used the probe to study the anti‐inflammation efficiency of nonsteroidal anti‐inflammatory drugs (NSAIDs, aspirin, diclofenac, and ibuprofen) in a carrageenan‐induced acute inflammation mouse model by measuring oxidative stress change. The authors discovered only inflamed mice treated with diclofenac showed significant fluorescence recovery in 0–150 min. The reason was that diclofenac impedes neutrophil recruitment to reduced MPO, further increasing intracellular RSS and fluorescence signals. Next, probe 43d was used to probe the oxidative stress in the CCl_4_‐induced liver injury and repair by NIR‐II in vivo imaging. To prove the superiorities of probe 43d, ICG was chosen for liver imaging as a control probe. Three groups of mice were treated with PBS, CCl_4_, and CCl_4_ + NAC, respectively, and then were imaged by ICG (808 nm‐channel) and 43d (980 nm‐channel) at the same time. The results demonstrated that both ICG and 43d could visualize the liver injury or liver repair with pronounced fluorescence decrease or increase, respectively. The author also found that ICG could be quickly cleared from the liver after 30 min while probe 43d could gradually enrich in the liver at 0–3 h in the control mouse. Both ICG and 43d displayed fluorescence decrease in the injury groups at 0–180 min (Figure 14C). In the injury and repair process, the 808 nm‐channel NIR‐II fluorescence signal in mouse livers monotonously decreased at 30–180 min even after the administration of NAC at 60 min; by contrast, the 980 nm‐channel fluorescence signals increased sharply at 120–180 min once the treatment of NAC was performed. The results clearly illustrated that 43d could real‐time monitor the dynamic changes of HClO and RSS in the consecutive liver injury and repair processes by reversible NIR‐II signal changes. It showed the first paradigm of in vivo studying the oxidative stress in liver injury‐repair processes with reversible NIR‐II fluorescence visualization, which may inspire more researchers to develop reversible NIR‐II probes for complicated disease processes.

#### 4.1.3 Probe for Enzymes and Metal Ions

**Figure 15:**
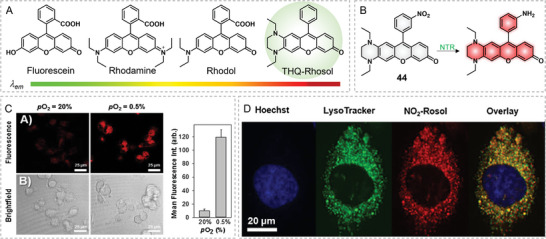
Structure of THQ–Rhosol‐based NTR probe. A) Development of THQ–Rhosol. Reproduced with permission.^[^ ^56^ ^]^ Copyright 2019, American Chemical Society. B) Reaction for probe 44 and NTR. C) Assessment of NTR activity in hypoxic cells. D) Lysosome‐localized property of probe 44. Reproduced with permission.^[^ ^57^ ^]^ Copyright 2020, Elsevier, B.V.

In 2019, Chin and Glass rationally created an uncharged NIR emissive Rhosol dye, THQ–Rhosol, for mapping tumor‐draining lymphatics by in vivo NIR imaging (**Figure** **15**A).^[^ ^56^ ^]^ Rhosol dyes can be regarded as an updated version of rhodol dyes, in which the pendant 2‐carboxyl benzene group is replaced with benzene; thus, lowering the pK_a_ of rhodol (from ≈6.3 to ≈4.8). Anyway, the fluorescence and Stokes shift of Rhosol were similar to that of conventional xanthene dyes, including fluorescein, rhodamine, and Rhodol. Through introducing THQ to the Rhosol scaffold, THQ–Rhosol displayed a NIR emission at 710 nm, a large Stokes shift (140 nm), a good photostability, and an appropriate pK_a_ of 5.8, making it become a good NIR contrast for visualizing tumor metastasis. After that, in 2020, Chin's group continuously reported a THQ–Rhosol‐based fluorescence probe 44 for nitroreductase (NTR) to assess hypoxia in tumorous cells (Figure 15B).^[^ ^57^ ^]^ Structurally, 3‐nitro‐benzene moiety could quench the fluorescence of probe 44 through donor excited photo‐induced electron transfer (*d*‐PET) effects. After being reduced by NTR, the 3‐nitrobenzene group turned to the 3‐aminobenzene group. The fluorescence at 705 nm was switched on with a maximal fluorescence increase of 28‐fold in solution due to the abolishment of *d*‐PET upon the reduction. The hypoxic cells displayed an 18‐fold increase in fluorescence signal than the normoxic cells with probe 44 (Figure 15C). 44 could accumulate well in lysosomes through cellular colocalization of the lysotracker dye and 44 (Figure 15D).

**Figure 16:**
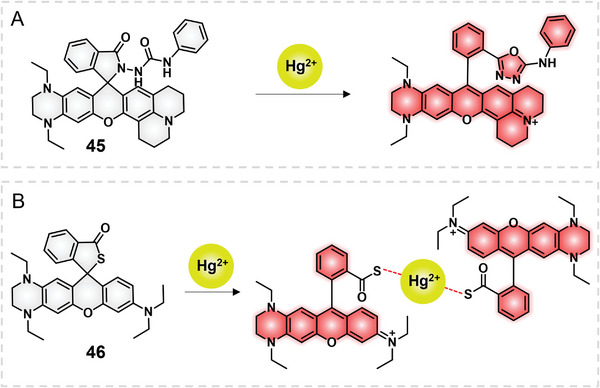
THQ–rhodamine based fluorescent probes for Hg^2+^. A) Structure of 45. B) Structure of 46.

Impelled by the THQ modification strategy, in 2020, Han and collaborators exploited a THQ–rhodamine‐based fluorescent probe 45 for mercury ion (Hg^2+^) detection in environmental water samples (**Figure** **16**A).^[^ ^58^ ^]^ The THQ modification could shift the emission of xanthene dyes to the NIR range and endow it with a large Stokes shift. On the other hand, the julolidine structure could promote the fluorescence quantum yield by impeding the TICT effect of an amino group.^[^ ^59^ ^]^ On these grounds, marrying THQ and julolidine groups with a xanthene scaffold would promote the NIR emission (664 nm), large Stokes shift, and fluorescence quantum yield together. The non‐fluorescent probe 45 was transferred to open‐ring rhodamine with high fluorescence. The probe showed a fast (<30 s) and sensitive (LOD = 1.87 ppb) response toward Hg^2+^ in tap water, lake water, and river water, holding good promise in Hg^2+^ detection in environmental water samples. Analogously, in 2020, Zeng's group reported a thioether spirocyclic THQ–rhodamine‐based NIR probe 46 for Hg^2+^ via.Hg^2+^‐promoted lactam ring open strategy (Figure 16B).^[^ ^60^ ^]^

### 4.2 Theranostic Applications

**Figure 17:**
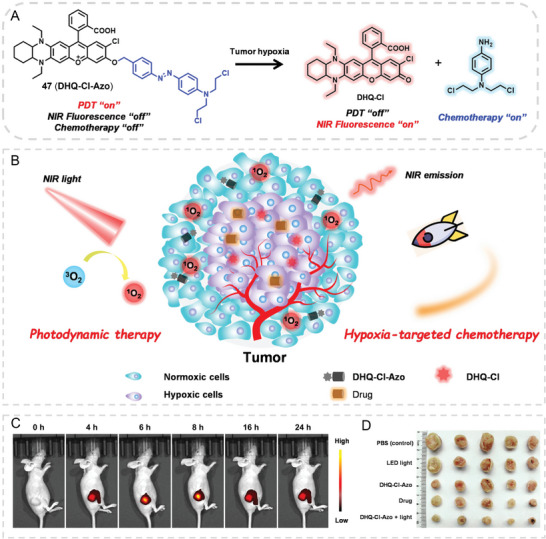
THQ–rhodol based theranostic platform for treating solid tumors. A) Working mechanism for 47. B) Illustration of 47‐based combined chemical and photodynamic therapies for tumors. C) Imaging tumors by 47. D) Therapeutic effects of 47 on solid tumors. Reproduced with permission.^[^ ^62^ ^]^ Copyright 2022, Elsevier, B.V.

THQ–xanthene dyes have been broadly used in bioimaging and biosensing, but trials of evolving THQ–xanthene‐based therapy for diseases and cancers have been rarely reported. Regarding the PDT/SDT/PPT applications of conventional xanthene dyes (eg. Rose Bengal and methylene blue),^[^ ^61^ ^]^ there is an increasing driving force to put THQ–xanthene dyes into further oncological therapy. With this regard, we embarked on using THQ–xanthene in theranostic applications. Luckily, in 2022, we and our collaborators reported the first THQ–xanthene‐based Azo reductase‐activated theranostic probe 47 for synergistically chemical and photodynamic therapies on solid tumors (**Figure** **17**A).^[^ ^62^ ^]^ Probe 47 was composited of three sections, a NIR emissive THQ–rhodol fluorophore, nitrogen mustard as the chemical drug, and an Azo‐reductase sensitive azo bond as the linker. In the probe, the NIR fluorescence of the probe was quenched by the azo‐aromatics and the drug activity of nitrogen mustard was inhibited due to azo modification on the drug. Surprisingly, probe 47 was type‐II PDT‐active and acted as a photosensitizer to catalyze oxygen to produce cytotoxic singlet oxygen (^1^O_2_) under light irradiation (Figure 17B). Through DFT calculations, the results demonstrated that the fluorophore moiety and the azo moiety of 47 were prone to form intramolecular enfoldment via *π*–*π* stacking in an aqueous solution, facilitating the ISC processes and enhancing PDT performance. After the reaction of azo reductase, the probe was reduced to release the NIR emissive and non‐photodynamic fluorophore and activated nitrogen mustard, realizing the tumor diagnosis and visual drug release. As the center of a solid tumor is hypoxic and its azo reductase is highly expressed, hypoxia activates the chemotherapy. In the out layer of a solid tumor, the oxygen content in tumor tissues is sufficient to run the PDT. As such, by taking advantage of the distinctive oxygen distribution of the out‐layer and center of solid tumors, the combination of different modular therapies (chemotherapy + PDT) can be performed at the different regions of solid tumors and produce a synergistic therapeutic index for solid tumors. In in vivo tumor therapy, the tumor growth was significantly suppressed after 20 days of therapy with the theranostic probe (Figure 17C,D). To sum up, this work proposed a novel tactic design for a molecular theranostic that enables the combined PDT and chemotherapy to cure solid tumors by taking the oxygen heterogenicity of tumors. The therapeutic idea can be extended to other therapies in preclinic and clinical tumor therapy.

### 4.3 Super‐Resolution Imaging Applications

**Figure 18:**
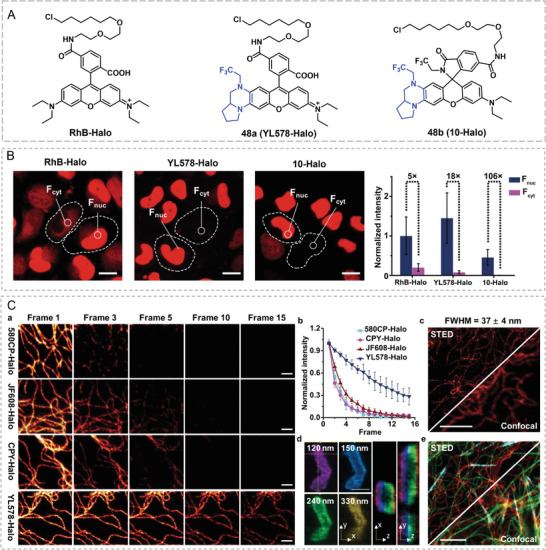
Super‐resolution imaging with THQ–rhodamine dyes. A) Structures of STED probes RhB‐Halo, 48a, and 48b. B) Fluorescence ratio of STED imaging with the Halo‐Tag probes. C) STED nanoscopic comparison of the Halo‐Tag probes. Reproduced with permission.^[^ ^34^ ^]^ Copyright 2022, Springer Nature Limited.

Beyond theranostic application, endeavors of using THQ–xanthene for super‐resolution imaging (SRI) have also been explored recently. Since the beginning of 2000, super‐resolution imaging has revolutionized fluorescence imaging with an unprecedented resolution of several to dozens of nanometers by breaking through the diffraction limit (200 nm) of conventional optical microscopy.^[^ ^63^ ^]^ In STED‐based super‐resolution imaging, a high‐power donut‐shaped depletion laser beam was used to decrease emission spot area; thus, rendering nanoscale resolution. As such, common dyes are encountered with serious photobleaching and only several dyes can be adopted in STED‐based super‐resolution imaging. Therefore, developing NIR fluorescent dyes with high brightness and photostability is imperative for STED nanoscopy. In 2022, Wang and Yuan jointly reported a series of THQ‐modified rhodamine‐based NIR dyes 48a/48b with a perfect balance of large Stokes shifts, brightness, and photostability, through introducing THQ moiety to rhodamine and grafting electron‐withdraw groups (trifluoroethyl) to the THQ part (**Figure** **18**A).^[^ ^34^ ^]^ The introduced THQ moiety could endow rhodamine dye 48a with a large Stokes shift (56 nm vs 27 nm) and a red‐shifted emission wavelength (634 nm vs 580 nm); and the grafted trifluoroethyl group could improve the fluorescence quantum yield and brightness through the prevention of the TICT formation. As such, the fluorescence quantum yield of 48a was much higher than that of rhodamine B (0.74 cf. 0.31). RhB‐Halo and 48a/48b could label HaloTag‐contained proteins in living cells without any washing procedures. Upon binding to HaloTag, 48b displayed a 490‐fold fluorescence increase due to the opening of lactam. The nuclei‐to‐cytosol signal ratios (*F*_nuc_ */F*_cyt_) of RhB‐Halo, 48a, and 48b were 5, 18, and 106, respectively, proving the cell staining abilities (Figure 18B). The authors further compared the STED‐nanoscopic performance of 48a and available STED dyes including 580CP‐Halo, CPY‐Halo, and JF608‐Halo. The full width at half maximum (FWHM) resolution for 580CP‐Halo, CPY‐Halo, and JF608‐Halo were 116 ± 6, 86 ± 9, and 83 ± 10 nm while the FWHM resolution of 48a was 37±4 nm in the first frame of cellular STED imaging. More importantly, the commercial dyes exhibited noticeable fluorescence quenching (>50% initial fluorescence intensity) after two to three frames of STED images, but 48a could provide enough bright super‐resolution imaging even after nine frames of STED images. In addition, 48a provided 3D STED images of mitochondria and three‐color STED images of vimentin filaments with other STED dyes in live cells (Figure 18C). The superior stability, brightness, and Stokes shifts contributed to the higher quality of cellular STED images with better nanoscale resolution in comparison to commercial STED dyes. This work proved the marvelous potential of THQ–xanthene dyes in super‐resolution imaging.

## 5 Conclusions and Future Outlook

In this review, we expounded on the advent, working principles, chemical structural modifications, and evolutional trajectory of THQ–xanthene dyes, including THQ–rhodamine, THQ–Rhodol, THQ–Rhosol, and other THQ–xanthene like dyes as well as the fluorescent probe‐based imaging and sensing and the potential theranostic and super‐resolution imaging applications with THQ‐xanthene dyes. With these achievements in the past 5 years, the THQ‐modification strategy has been solidly substantiated to be an accessible, effective, and universal method to improve xanthene dyes with NIR‐I/II emissions and large Stokes shifts. The THQ modification strategy for xanthene is powerful and appealing, that is, a minor structural modification sparked great improvements in Stokes shift and NIR‐I/II emission. Without a doubt, this strategy would enrich the toolbox of chemical, biological, and medical science researchers to create desirable NIR‐I/II fluorophores for a broad spectrum of fluorescence‐based applications, ranging from the detection of biological and environmental active species to imaging‐guided cancer therapies.

As an emerging and burgeoning tactic to design NIR‐I/II xanthene, this strategy also has much‐unresearched areas:

1) The universality of this strategy to other common fluorophores should be fully investigated as much as possible. Apart from xanthene dyes, can the THQ modification strategy apply to other classic fluorophores and dyes, such as cyanine, coumarin, BODIPY, AIEgen, and benzobisthiadiazole (BBD)‐based fluorophores with improved NIR emission wavelengths and Stokes shifts? Luckily, the THQ modified coumarin dyes also displayed NIR emissions and large Stokes shifts.^[^ ^64^ ^]^ In addition, several THQ‐modified hybrid dyes of rhodamine and hemi‐cyanine preliminary confirmed the feasibility of the approach. So far, no THQ‐modified BODIPY, AIEgen, or BBD‐based NIR‐II fluorophore has yet been reported. Beyond them, THQ‐modified symmetric structural dyes should also be fabricated and studied, which is an unexplored area for THQ‐modified dyes. Overall, comprehensively studying the validity of the THQ modification to a diversity of dyes should be further studied with enough sample sizes.
2) How to increase the brightness of THQ–xanthene dyes is critical for bioimaging. Frankly, the fluorescence quantum yields of THQ–xanthene dyes are generally moderate compared with their parent dyes. To enhance fluorescence quantum yields, THQ–xanthene dyes with more rigid and coplanar *π*‐system (dyes 11 and 13), rational halogen substitution (dyes 22–24), and electron‐withdraw substitutes on the nitrogen of THQ (dyes 27–31) have been proven to work effectively.^[^ ^32^, ^34^, ^65^ ^]^ Further, strategies reducing the *π*–*π* stacking of these fluorophores and obstructing interactions between water molecules and fluorophores via bulky alkyl substitutes may also boost the emission efficiency^[^ ^66^ ^]^ but has not proved in THQ‐xanthene dyes yet. In the case of absorption efficiency, elongating the *π*‐conjugated system and introducing a cationic *π*‐system are representative and powerful approaches.^[^ ^67^ ^]^
3) One blemish of the THQ modification method is that the absorption of THQ–xanthene dyes cannot be efficiently bathochromic‐shifted to the NIR absorption range. For instance, the NIR emissive THQ–rhodamine and THQ–rhodol usually have absorption bands centered at the ≈560–600 nm range, almost similar to their parent rhodamine and rhodol (Table 1). For in vivo imaging and phototherapies, NIR absorption and NIR emission are both essential. Thereupon, THQ modification is often required to coordinate with other strategies including *π*‐expansion and ten‐substitution methods to balance NIR‐I/II absorbed and emissive xanthene dyes synergistically.
4) Beyond fluorescence imaging, extending THQ‐xanthene in therapeutic applications should be deeply explored. Due to the short history of THQ–xanthene since 2017, most THQ–xanthene dyes have been intensely deployed in fluorescence‐based imaging and detection, and only one report about THQ‐xanthene based theranostic was used for cancer imaging and therapy by combining photodynamic therapy (PDT) and chemotherapy.^[^ ^62^ ^]^ In this situation, we thirsted for if THQ–xanthene could be further used in phototherapies (PDT and PTT), sonodynamic therapy, disease and cancer theranostics, and imaging‐guided surgery;^[^ ^67^, ^68^ ^]^ thus, benefiting the preclinical and clinical applications of THQ–xanthene dyes.

To reach these goals, more efforts from us and other scientists should be dedicated to this burgeoning area of THQ–xanthene dyes.

## Conflict of Interest

The authors declare no conflict of interest.

## References

[1] a) H. Li , H. Kim , F. Xu , J. Han , Q. Yao , J. Wang , K. Pu , X. Peng , J. Yoon , Chem. Soc. Rev. 2022, 51, 1795; 35142301 10.1039/d1cs00307k

[2] a) Y. Liu , Y. Li , S. Koo , Y. Sun , Y. Liu , X. Liu , Y. Pan , Z. Zhang , M. Du , S. Lu , X. Qiao , J. Gao , X. Wang , Z. Deng , X. Meng , Y. Xiao , J. S. Kim , X. Hong , Chem. Rev. 2022, 122, 209; 34664951 10.1021/acs.chemrev.1c00553

[3] a) X. Ge , Y. Lou , L. Su , B. Chen , Z. Guo , S. Gao , W. Zhang , T. Chen , J. Song , H. Yang , Anal. Chem. 2020, 92, 6111; 32216270 10.1021/acs.analchem.0c00556

[4] a) D. Song , C. Li , M. Zhu , S. Chi , Z. Liu , Angew. Chem., Int. Ed. 2022, 61, e202212721; 10.1002/anie.202212721 36123304

[5] a) Z. Zeng , S. S. Liew , X. Wei , K. Pu , Angew. Chem., Int. Ed. 2021, 60, 26454; 10.1002/anie.202107877 34263981

[6] a) J. B. Grimm , L. D. Lavis , Nat. Methods 2022, 19, 149; 34949811 10.1038/s41592-021-01338-6

[7] X. Chen , T. Pradhan , F. Wang , J. S. Kim , J. Yoon , Chem. Rev. 2012, 112, 1910. 22040233 10.1021/cr200201z

[8] a) O. Karaman , G. A. Alkan , C. Kizilenis , C. C. Akgul , G. Gunbas , Coord. Chem. Rev. 2023, 475, 214841;

[9] a) L. Wang , W. Du , Z. Hu , K. Uvdal , L. Li , W. Huang , Angew. Chem., Int. Ed. 2019, 58, 14026; 10.1002/anie.201901061 30843646

[10] a) G. Niu , P. Zhang , W. Liu , M. Wang , H. Zhang , J. Wu , L. Zhang , P. Wang , Anal. Chem. 2017, 89, 1922; 28208300 10.1021/acs.analchem.6b04417

[11] Z. Lei , X. Li , X. Luo , H. He , J. Zheng , X. Qian , Y. Yang , Angew. Chem., Int. Ed. 2017, 56, 2979. 10.1002/anie.201612301 28140490

[12] D. Liu , Z. He , Y. Zhao , Y. Yang , W. Shi , X. Li , H. Ma , J. Am. Chem. Soc. 2021, 143, 17136. 34632770 10.1021/jacs.1c07711

[13] a) H. Bian , D. Ma , F. Pan , X. Zhang , K. Xin , X. Zhang , Y. Yang , X. Peng , Y. Xiao , J. Am. Chem. Soc. 2022, 144, 22562; 36445324 10.1021/jacs.2c08602

[14] a) H. N. Kim , M. H. Lee , H. J. Kim , J. S. Kim , J. Yoon , Chem. Soc. Rev. 2008, 37, 1465; 18648672 10.1039/b802497a

[15] a) M. Fu , Y. Xiao , X. Qian , D. Zhao , Y. Xu , Chem. Commun. 2008, 1780, 10.1039/B718544H; 18379691

[16] a) Y. Koide , Y. Urano , K. Hanaoka , W. Piao , M. Kusakabe , N. Saito , T. Terai , T. Okabe , T. Nagano , J. Am. Chem. Soc. 2012, 134, 5029; 22390359 10.1021/ja210375e

[17] a) Y. Koide , Y. Urano , K. Hanaoka , T. Terai , T. Nagano , ACS Chem. Biol. 2011, 6, 600; 21375253 10.1021/cb1002416

[18] a) X. Zhou , R. Lai , J. R. Beck , H. Li , C. I. Stains , Chem. Commun. 2016, 52, 12290; 10.1039/c6cc05717a PMC5108567 27709196

[19] Z. Ding , C. Wang , M. Fan , M. Zhang , Y. Zhou , X. Cui , D. Zhang , T. Wang , Chem. Commun. 2020, 56, 13579. 10.1039/d0cc05529h 33052367

[20] J. Liu , Y.‐Q. Sun , H. Zhang , H. Shi , Y. Shi , W. Guo , ACS Appl. Mater. Interfaces 2016, 8, 22953. 27548811 10.1021/acsami.6b08338

[21] a) Q. A. Best , N. Sattenapally , D. J. Dyer , C. N. Scott , M. E. McCarroll , J. Am. Chem. Soc. 2013, 135, 13365; 23889259 10.1021/ja401426s

[22] J. B. Grimm , T. A. Brown , A. N. Tkachuk , L. D. Lavis , ACS Cent. Sci. 2017, 3, 975. 28979939 10.1021/acscentsci.7b00247 PMC5620978

[23] a) A. Choi , S. C. Miller , Org. Lett. 2018, 20, 4482; 30014702 10.1021/acs.orglett.8b01786 PMC6524783

[24] a) M. Beija , C. A. M. Afonso , J. M. G. Martinho , Chem. Soc. Rev. 2009, 38, 2410; 19623358 10.1039/b901612k

[25] a) J. Xiong , W. Wang , C. Wang , C. Zhong , R. Ruan , Z. Mao , Z. Liu , ACS Sens. 2020, 5, 3237; 33092345 10.1021/acssensors.0c01555

[26] X. Zhang , T. Ren , F. Yang , L. Yuan , Chin. Chem. Lett. 2021, 32, 3890.

[27] C. Wang , W. Chi , Q. Qiao , D. Tan , Z. Xu , X. Liu , Chem. Soc. Rev. 2021, 50, 12656. 34633008 10.1039/d1cs00239b

[28] W. Chen , S. Xu , J. J. Day , D. Wang , M. Xian , Angew. Chem., Int. Ed. 2017, 56, 16611. 10.1002/anie.201710688 PMC5773255 29134784

[29] J. B. Grimm , B. P. English , J. Chen , J. P. Slaughter , Z. Zhang , A. Revyakin , R. Patel , J. J. Macklin , D. Normanno , R. H. Singer , T. Lionnet , L. D. Lavis , Nat. Methods 2015, 12, 244. 25599551 10.1038/nmeth.3256 PMC4344395

[30] T.‐B. Ren , W. Xu , W. Zhang , X.‐X. Zhang , Z.‐Y. Wang , Z. Xiang , L. Yuan , X.‐B. Zhang , J. Am. Chem. Soc. 2018, 140, 7716. 29792690 10.1021/jacs.8b04404

[31] B. Li , M. Zhao , F. Zhang , ACS Mater. Lett. 2020, 2, 905.

[32] P. Wang , L. Yu , J. Gong , J. Xiong , S. Zi , H. Xie , F. Zhang , Z. Mao , Z. Liu , J. S. Kim , Angew. Chem., Int. Ed. 2022, 61, e202206894. 10.1002/anie.202206894 35789171

[33] W. Chi , Q. Qiao , C. Wang , J. Zheng , W. Zhou , N. Xu , X. Wu , X. Jiang , D. Tan , Z. Xu , X. Liu , Angew. Chem., Int. Ed. 2020, 59, 20215. 10.1002/anie.202010169 32776641

[34] G. Jiang , T.‐B. Ren , E. D'Este , M. Xiong , B. Xiong , K. Johnsson , X.‐B. Zhang , L. Wang , L. Yuan , Nat. Commun. 2022, 13, 2264. 35477933 10.1038/s41467-022-29547-3 PMC9046415

[35] a) X. Song , A. Johnson , J. Foley , J. Am. Chem. Soc. 2008, 130, 17652; 19108696 10.1021/ja8075617

[36] a) Z. Ye , W. Yang , C. Wang , Y. Zheng , W. Chi , X. Liu , Z. Huang , X. Li , Y. Xiao , J. Am. Chem. Soc. 2019, 141, 14491; 31487156 10.1021/jacs.9b04893

[37] a) Q. Yang , Z. Ma , H. Wang , B. Zhou , S. Zhu , Y. Zhong , J. Wang , H. Wan , A. Antaris , R. Ma , X. Zhang , J. Yang , X. Zhang , H. Sun , W. Liu , Y. Liang , H. Dai , Adv. Mater. 2017, 29, 1605497; 10.1002/adma.201605497 28117499

[38] A. Ji , H. Lou , C. Qu , W. Lu , Y. Hao , J. Li , Y. Wu , T. Chang , H. Chen , Z. Cheng , Nat. Commun. 2022, 13, 3815. 35780137 10.1038/s41467-022-31521-y PMC9250501

[39] a) R. Tian , Q. Zeng , S. Zhu , J. Lau , S. Chandra , R. Ertsey , K. S. Hettie , T. Teraphongphom , Z. Hu , G. Niu , D. O. Kiesewetter , H. Sun , X. Zhang , A. L. Antaris , B. R. Brooks , X. Chen , Sci. Adv. 2019, 5, eaaw0672; 31548981 10.1126/sciadv.aaw0672 PMC6744268

[40] a) J. Huang , K. Pu , Angew. Chem., Int. Ed. 2020, 59, 11717; 10.1002/anie.202001783 32134156

[41] T.‐B. Ren , Z.‐Y. Wang , Z. Xiang , P. Lu , H.‐H. Lai , L. Yuan , X.‐B. Zhang , W. Tan , Angew. Chem., Int. Ed. 2021, 60, 800. 10.1002/anie.202009986 32918358

[42] Z. Qin , T.‐B. Ren , H. Zhou , X. Zhang , L. He , Z. Li , X.‐B. Zhang , L. Yuan , Angew. Chem., Int. Ed. 2022, 61, e202201541. 10.1002/anie.202201541 35218130

[43] V. S. Lin , W. Chen , M. Xian , C. J. Chang , Chem. Soc. Rev. 2015, 44, 4596. 25474627 10.1039/c4cs00298a PMC4456340

[44] X. Zhang , H. Liu , Y. Ma , W. Qu , H. He , X. Zhang , S. Wang , Q. Sun , F. Yu , Dyes Pigm. 2019, 171, 107722.

[45] J. Hong , Q. Xia , E. Zhou , G. Feng , Talanta 2020, 215, 120914. 32312458 10.1016/j.talanta.2020.120914

[46] Z. Mao , L. Hu , X. Dong , C. Zhong , B.‐F. Liu , Z. Liu , Anal. Chem. 2014, 86, 6548. 24877642 10.1021/ac501947v

[47] Y. Zhang , S. Xia , M. Fang , W. Mazi , Y. Zeng , T. Johnston , A. Pap , R. L. Luck , H. Liu , Chem. Commun. 2018, 54, 7625. 10.1039/c8cc03520b PMC6058674 29927444

[48] Z. Mao , J. Xiong , P. Wang , J. An , F. Zhang , Z. Liu , J. Seung Kim , Coord. Chem. Rev. 2022, 454, 214356.

[49] Q. Xia , S. Feng , J. Hong , G. Feng , Sens. Actuators, B 2021, 337, 129732.

[50] S. Ma , G. Chen , J. Xu , Y. Liu , G. Li , T. Chen , Y. Li , T. D. James , Coord. Chem. Rev. 2021, 427, 213553.

[51] a) P. Verwilst , H. S. Kim , S. Kim , C. Kang , J. S. Kim , Chem. Soc. Rev. 2018, 47, 2249; 29484335 10.1039/c7cs00706j

[52] a) C. A. Lipinski , F. Lombardo , B. W. Dominy , P. J. Feeney , Adv. Drug Delivery Rev. 1997, 23, 3; 10.1016/s0169-409x(00)00129-0 11259830

[53] a) Y. Wen , N. Jing , M. Zhang , F. Huo , Z. Li , C. Yin , Adv. Sci. 2023, 10, 2206681; 10.1002/advs.202206681 PMC10015879 36651112

[54] L. He , L.‐H. He , S. Xu , T.‐B. Ren , X.‐X. Zhang , Z.‐J. Qin , X.‐B. Zhang , L. Yuan , Angew. Chem., Int. Ed. 2022, 61, e202211409. 10.1002/anie.202211409 36149874

[55] S. S. Matikonda , G. Hammersley , N. Kumari , L. Grabenhorst , V. Glembockyte , P. Tinnefeld , J. Ivanic , M. Levitus , M. J. Schnermann , J. Org. Chem. 2020, 85, 5907. 32275153 10.1021/acs.joc.0c00236 PMC8459201

[56] K. S. Hettie , J. L. Klockow , T. E. Glass , F. T. Chin , Anal. Chem. 2019, 91, 3110. 30669835 10.1021/acs.analchem.8b05709 PMC6516061

[57] J. L. Klockow , K. S. Hettie , E. L. LaGory , E. J. Moon , A. J. Giaccia , E. E. Graves , F. T. Chin , Sens. Actuators, B 2020, 306, 127446. 10.1016/j.snb.2019.127446 PMC7138224 32265579

[58] S. Chen , S. Zhang , A. Ruhan , Y. Han , Tetrahedron Lett. 2020, 61, 152077.

[59] R. J. Iwatate , M. Kamiya , K. Umezawa , H. Kashima , M. Nakadate , R. Kojima , Y. Urano , Bioconjugate Chem. 2018, 29, 241. 10.1021/acs.bioconjchem.7b00776 29323873

[60] J. Gong , C. Liu , X. Jiao , S. He , L. Zhao , X. Zeng , RSC Adv. 2020, 10, 29536. 35521149 10.1039/d0ra04373g PMC9055982

[61] a) H. Lei , J. H. Kim , S. Son , L. Chen , Z. Pei , Y. Yang , Z. Liu , L. Cheng , J. S. Kim , ACS Nano 2022, 16, 10979; 35723442 10.1021/acsnano.2c03395

[62] J. Xiong , P. Wang , S. Son , C. Zhong , F. Zhang , Z. Mao , Z. Liu , J. S. Kim , Matter 2022, 5, 1502.

[63] H. Fang , Y. Chen , S. Geng , S. Yao , Z. Guo , W. He , Anal. Chem. 2022, 94, 17904. 36480812 10.1021/acs.analchem.2c03887

[64] a) L. He , H. Xiong , B. Wang , Y. Zhang , J. Wang , H. Zhang , H. Li , Z. Yang , X. Song , Anal. Chem. 2020, 92, 11029; 32697911 10.1021/acs.analchem.0c00030

[65] G. Jiang , X.‐F. Lou , S. Zuo , X. Liu , T.‐B. Ren , L. Wang , X.‐B. Zhang , L. Yuan , Angew. Chem., Int. Ed. 2023, 62, e202218613. 10.1002/anie.202218613 36855015

[66] R. Tian , H. Ma , S. Zhu , J. Lau , R. Ma , Y. Liu , L. Lin , S. Chandra , S. Wang , X. Zhu , H. Deng , G. Niu , M. Zhang , A. L. Antaris , K. S. Hettie , B. Yang , Y. Liang , X. Chen , Adv. Mater. 2020, 32, 1907365. 10.1002/adma.201907365 32022975

[67] a) X.‐Y. Ran , P. Chen , Y.‐Z. Liu , L. Shi , X. Chen , Y.‐H. Liu , H. Zhang , L.‐N. Zhang , K. Li , X.‐Q. Yu , Adv. Mater. 2023, 35, 2210179; 10.1002/adma.202210179 36630669

[68] a) X. Ni , X. Zhang , X. Duan , H.‐L. Zheng , X.‐S. Xue , D. Ding , Nano Lett. 2019, 19, 318; 30556699 10.1021/acs.nanolett.8b03936

